# Reactive Sintering Mechanism and Phase Formation in Ni-Ti-Al Powder Mixture During Heating

**DOI:** 10.3390/ma11050689

**Published:** 2018-04-27

**Authors:** Pavel Salvetr, Andrea Školáková, Cyprien Hudrisier, Pavel Novák, Dalibor Vojtěch

**Affiliations:** Department of Metals and Corrosion Engineering, University of Chemistry and Technology, Prague, Technická 5, 16628 Prague, Czech Republic; andrea.skolakova@vscht.cz (A.Š.); cyprien.hud@orange.fr (C.H.); panovak@vscht.cz (P.N.); vojtechd@vscht.cz (D.V.)

**Keywords:** intermetallics, reactive sintering, self-propagating high-temperature synthesis (SHS), Ni-Ti-Al alloy, differential thermal analysis

## Abstract

This work aims to describe the formation of intermetallics in the Ni-Ti-Al system in dependence on the heating rate, which has been determined previously as the crucial factor of thermal explosion self-propagating synthesis (TE-SHS). The tested alloys contained 1–7 wt % aluminum. Thermal analysis has been realized by the optical pyrometer under the conditions of high heating rates up to 110 °C·min^−1^. TE-SHS process in Ni-Ti-Al system is initiated by exothermic reaction of nickel aluminides Ni_2_Al_3_ and NiAl_3_ at the temperature of 535–610 °C. The next reactions occur in dependence on the heating rate. Samples containing 1–3 wt % of aluminum exhibit the similar initiation temperature as Ni-Ti binary mixture. The samples containing 5 wt % and more of aluminum were fully reacted after sintering at 800 °C with the heating rate of 300 °C·min^−1^ and the initiation temperature of the TE-SHS was observed close to Al-Al_3_Ni eutectic temperature (between 630–640 °C).

## 1. Introduction

Some binary and ternary intermetallic compounds based on nickel, aluminum, titanium, and iron excel in properties like good mechanical properties, shape memory, thermal stability, and also low density. For example Ti-Al alloys with low density and at the same time high mechanical properties are appropriate for aerospace industry. The addition of silicon improves the oxidation resistance at high temperatures [1]. The approximately equimolar Ni-Ti alloy is a well-known shape memory material. The shape memory effects are caused by non-diffusion phase transformation between austenite (high-temperature cubic structure) and martensite (low-temperature monoclinic structure) in the NiTi intermetallic phase. Intermetallics are usually produced by conventional melting and casting methods such as vacuum induction melting (VIM) and vacuum arc re-melting (VAR) [2]. These production methods are expensive and problematic due to high reactivity of melt and poor casting properties. The carbon crucibles react with the melt. Therefore the special crucibles coated by zirconia or yttria must be used for Ni-Ti alloys and the production price grows [3]. Powder metallurgy offers perspective methods of production of Ni-Ti alloys and other intermetallic compounds [2,4,5,6,7,8]. The elemental powders are mixed and pressed and consequently the pressed powder mixture compact is heated to the ignition temperature (which is lower than the melting point). The exothermic combustion reaction is initiated and leads to the formation of intermetallic phases. If the whole compressed compact is heated at the same time, the process is called ‘thermal explosion mode of self-propagating high-temperature synthesis’ (TE-SHS). If the heat source is focused only on one side of the compressed powder mixture compact and the reaction goes through the compact gradually, the mode of SHS reaction is called ‘plane wave propagation mode’ [9]. In the binary Ni-Ti system, the eutectic temperature 942 °C has been stated to be the initial temperature of the SHS reaction in some publications [10]. The initial temperature of approximately 890 °C was found out and the start of reaction is connected with α→β titanium transformation at the temperature of 882 °C [7]. The influence of heating rate, temperature of reactive sintering, particle sizes of nickel and titanium powders on microstructure, phase composition, and porosity were investigated in many publications [4,10,11,12,13]. 

The addition of aluminum into the near equiatomic Ni-Ti powder mixture was researched and the decrease of porosity and improvement of corrosion resistance and hardness were reported [14]. The two exothermic peaks were measured during heating of Ni-Ti-Al pressed compact containing 10–40 at % of aluminum. The first peak was observed at the temperature range of 595–625 °C, being related to the reaction between nickel and aluminum at eutectic point at the temperature of 640 °C. The second exothermic peak accompanies the reaction between nickel and titanium in the temperature range 938–946 °C at approximately eutectic point. Increasing amount of aluminum leads to higher exothermicity of the first peak and to lower exothermicity of the second peak [15].

In the present study, the addition of a low amount of aluminum (1–7 wt %) into the near equiatomic Ni-Ti powder mixture is studied. The mechanism of the reactions was studied in dependence of the heating rate.

## 2. Results and Discussion

**Figure 1:**
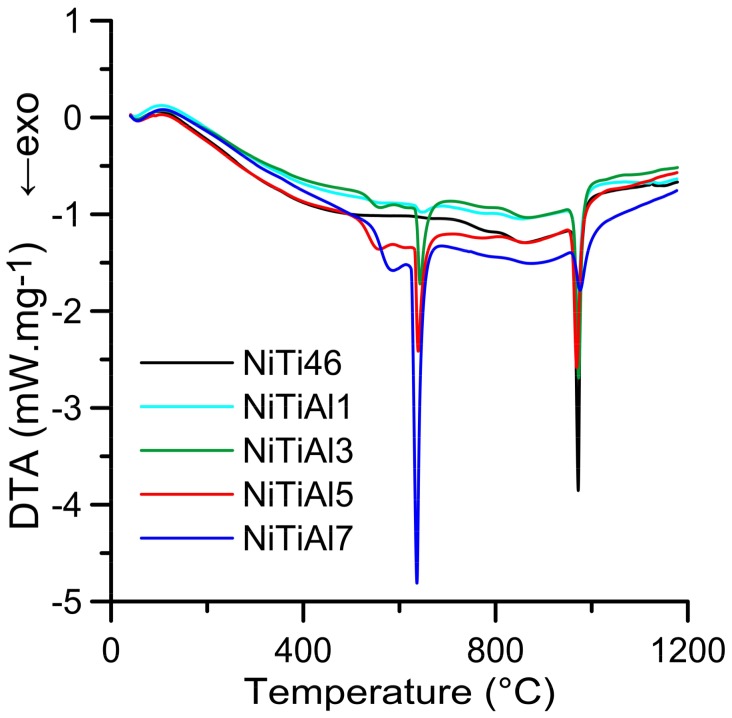
DTA heating curves of NiTi46 and NiTiAl1––7 to 1200 °C.

**Figure 2:**
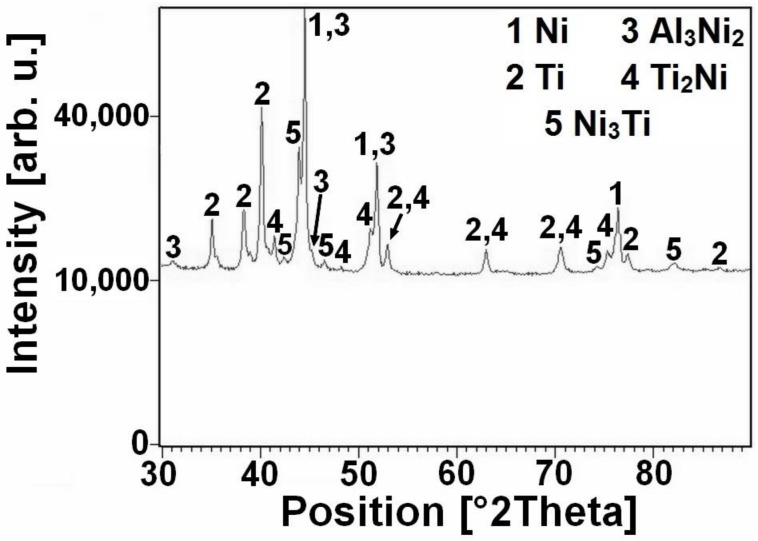
XRD pattern of NiTiAl5 sintered at 610 °C with duration of 20 min and quickly cooled in water.

The DTA heating curves of the equimolar NiTi46 and NiTiAl powder mixtures with various content of aluminum (1–7 wt %) are presented in Figure 1. There is only one strong exothermic peak with the onset temperature at 949 °C in the binary NiTi46 alloy. This peak corresponds with the temperature of the eutectic reaction (β-Ti + Ti_2_Ni → L) at 942 °C, which has been reported recently as the ignition point of the TE-SHS reaction [10]. The new exothermic double-peak appeared below the melting point of aluminum. The first part of the peak (535–610 °C) represents formation of the nickel aluminides-Ni_2_Al_3_, the Ni_3_Ti and Ti_2_Ni phases were formed due to solid state reaction, moreover unreacted nickel and titanium particles were identified by XRD patterns NiTiAl5 powder mixtures heated to 610 °C for 20 min (see in Figure 2) and these XRD results are in agreement with previous publications [15]. The strongly exothermic part of this peak (onset at approximately 632 °C) represents the TE-SHS reaction in the NiTiAl5 and NiTiAl7 powder mixture. The amount of 1 and 3 wt % of aluminum in powder mixture is not ample for initiation of reaction in the whole powder mixture. There is only a very weak exothermic peak in the NiTiAl1 sample detected at 633 °C.

**Figure 3:**
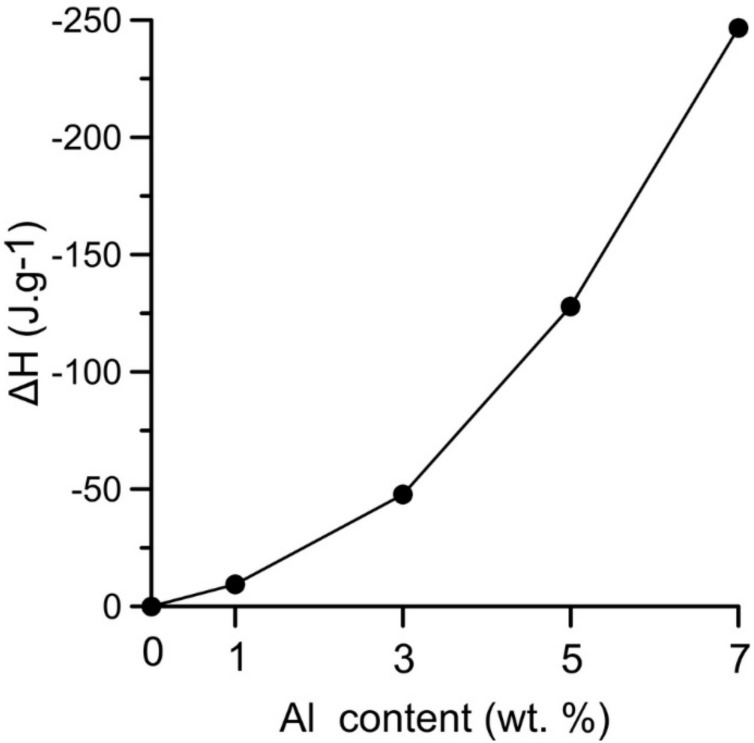
Relative heat associated with reaction at 535–650 °C depending on aluminum content in powder mixture.

The heat generated by the solid state reaction can lead to local formation of eutectic melt and to a rapid a strong support of the occurring reaction which represented the second part of the double-peak starting at the temperature interval 615–635 °C (the initiation temperature decreases with increasing aluminum content). The relative heat release (ΔH) associated with the exothermic reactions occurring in the range of 535–650 °C increases with aluminum content, see in Figure 3. The significant exothermic peak appeared in samples with the addition of aluminum in the amount of 3–7 wt %. However, the heat released by these reactions is not sufficient for reaction in the whole NiTiAl3 powder mixture.

**Figure 4:**
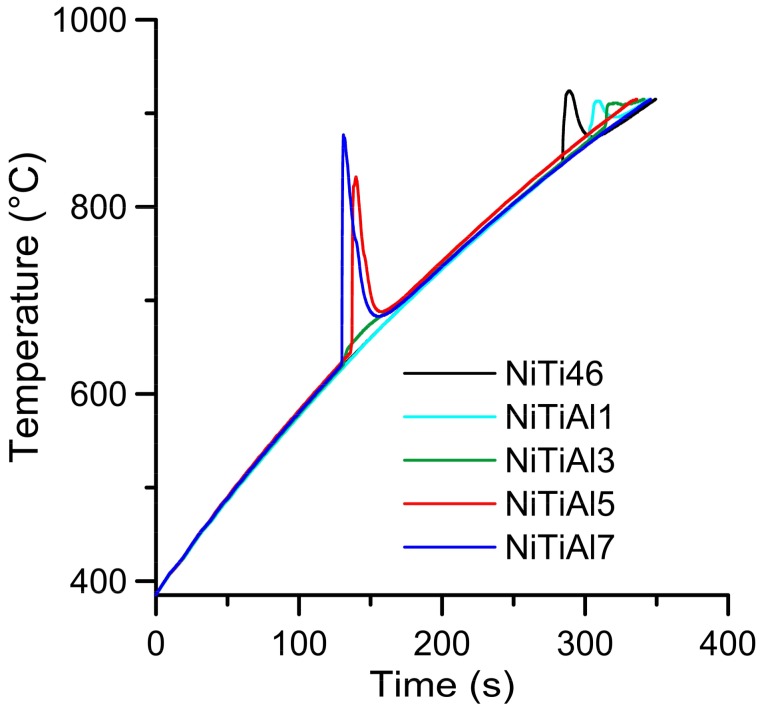
Heating curves of NiTi46 and NiTiAl1–7, recorded by optical pyrometer.

The similar phase evolution was observed in publication [15], the little shift of the measured peak’s temperature is caused by the use of a lower DSC (differential scanning calorimetry) heating rate (20 °C·min^−1^). The exothermic reaction close to temperatures of 942–955 °C is similar to the eutectic reaction in the binary Ni-Ti system. The influence of heating rate was studied in publication [4] and it was found that the higher heating rate leads to lower content of the Ti_2_Ni phase in the sintered product. The combination of induction furnace and optical pyrometer was used as the alternative way to DTA (differential thermal analysis), enabling observation of thermal effects during the heating with the higher heating rates of approximately 100 °C·min^–1^. The initiation temperature of 863 °C was measured in the NiTi46 alloy and the addition of 1 and 3 wt % of aluminum slightly increased this temperature (NiTiAl1–869 °C, NiTiAl3–885 °C). The reason for this effect consists in partial reaction around the elemental particles at the temperature of 632 °C. This reaction is possible to see in a heating curve recorded by optical pyrometer in the sample NiTiAl3, where is the minor peak at 632 °C (Figure 4).

**Figure 5:**
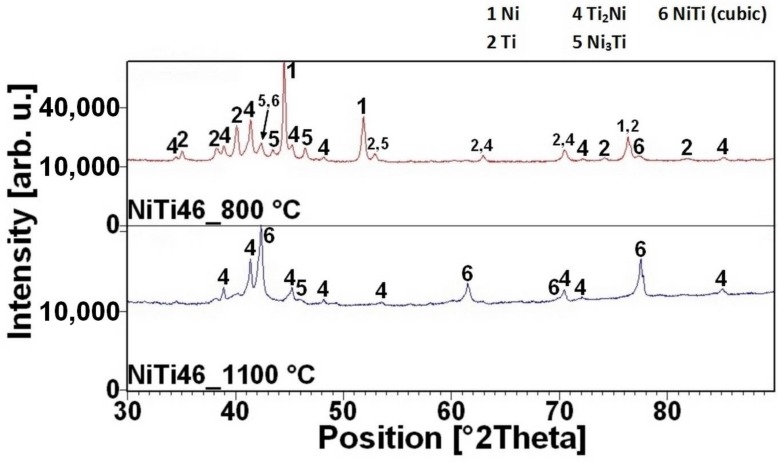
XRD patterns of the NiTi46 sample sintered at 800 and 1100 °C.

**Figure 6:**
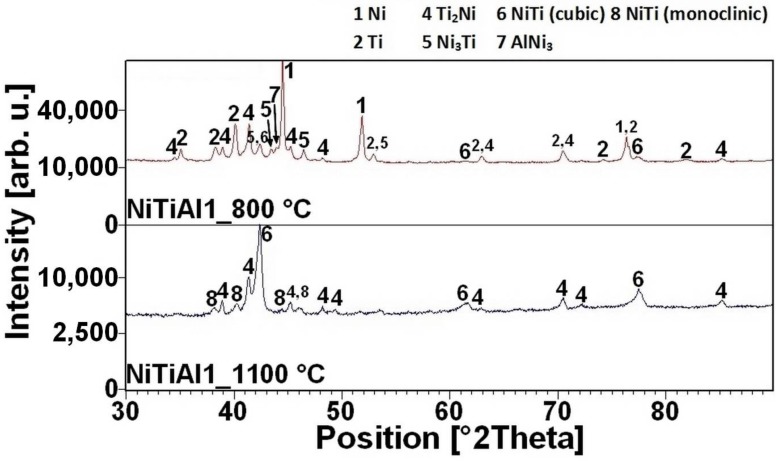
XRD patterns of the NiTiAl1 sample sintered at 800 and 1100 °C.

**Figure 7:**
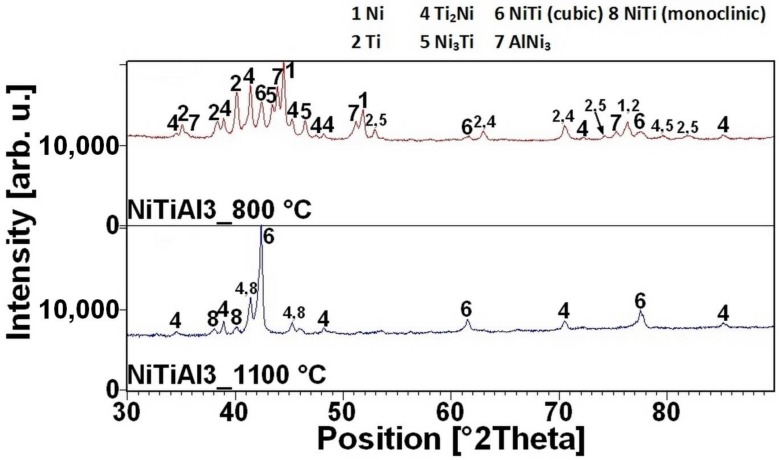
XRD patterns of the NiTiAl3 sample sintered at 800 and 1100 °C.

**Figure 8:**
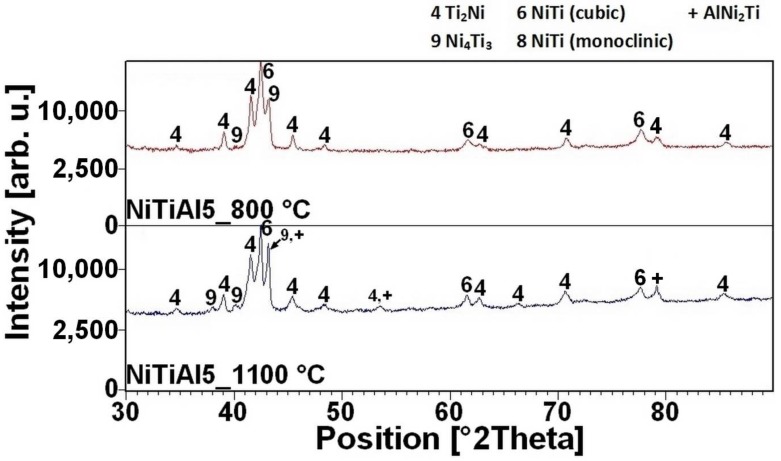
XRD patterns of the NiTiAl5 sample sintered at 800 and 1100 °C.

**Figure 9:**
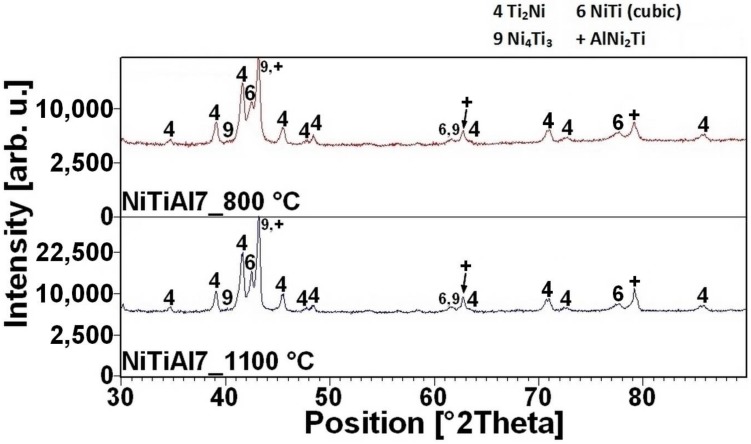
XRD patterns of the NiTiAl7 sample sintered at 800 and 1100 °C.

The reacted layer suppresses the TE-SHS at higher temperatures. In the NiTiAl3 sample, there the aluminides and Ti_2_Ni layer are thicker and, therefore, the TE-SHS starts at higher temperatures than in the NiTiAl1 sample. A similar effect prevents the possibility to sinter elemental Ni-Ti powder mixture in spark plasma sintering (SPS) [16]. There is the initiation temperature at 632 °C from 5 to 7 wt % of aluminum addition into powder mixture. The heating curves of the DTA and optical pyrometer are in good agreement with NiTiAl samples, and the initiation temperature of TE-SHS reaction was measured below the 882 °C (the α → β Ti) in the NiTi46 powder mixture. The results of XRD analysis for NiTi46 and NiTiAl1-7 samples sintered at 800 and 1100 °C with the heating rate of approximately 300 °C·min^−1^ are stated in Figure 5, Figure 6, Figure 7, Figure 8 and Figure 9 and they correspond with measuring in induction furnace and pyrometer because TE-SHS was completed fully at 800 °C in the samples with 5 and 7 wt % of aluminum.

**Figure 10:**
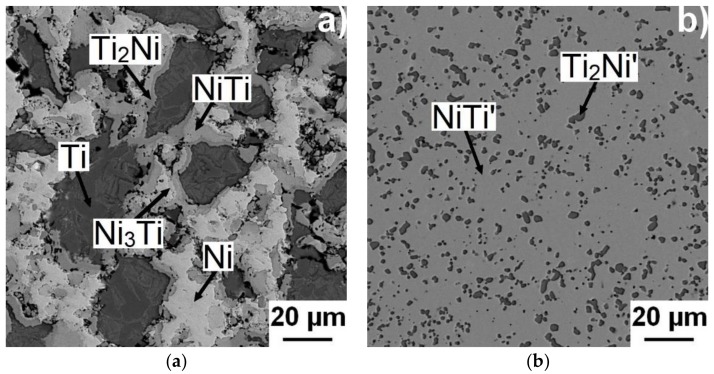

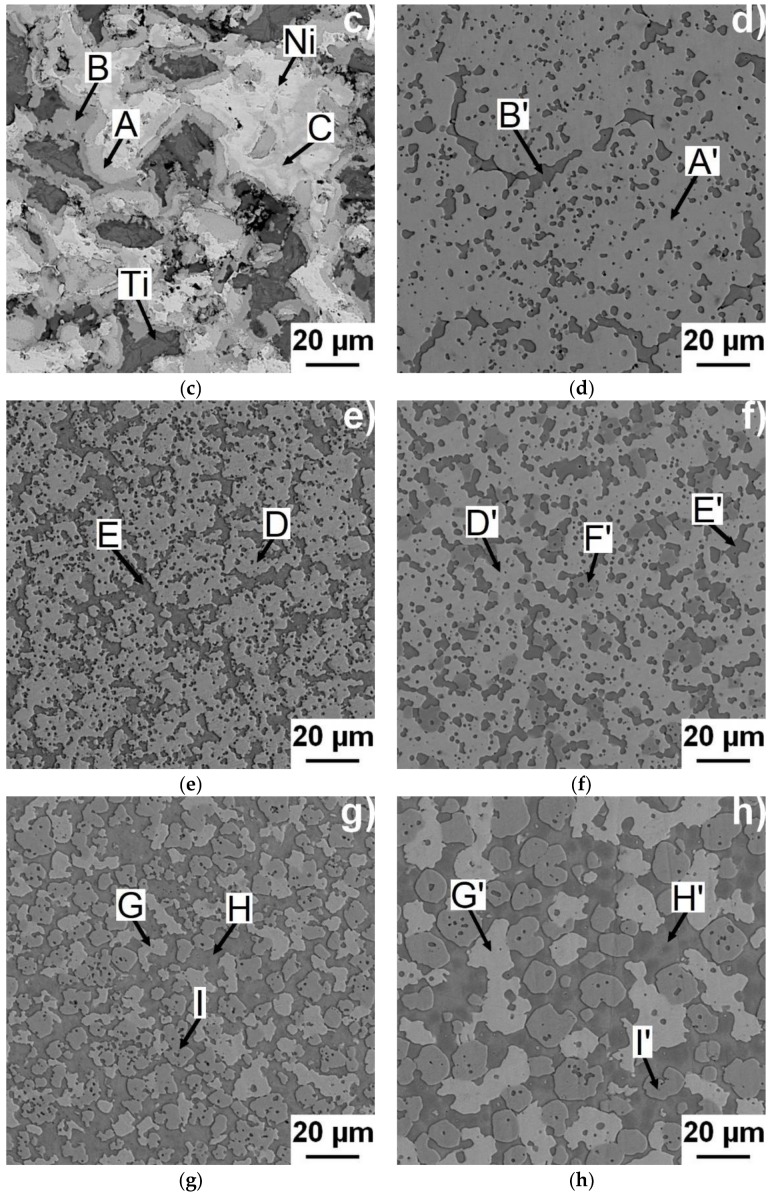
Microstructures of NiTi46 sintered at (**a**) 800 °C and (**b**) 1100 °C; NiTiAl3 at (**c**) 800 °C and (**d**) 1100 °C; NiTiAl5 at (**e**) 800 °C and (**f**) 1100 °C; and NiTiAl7 at (**g**) 800 °C and (**h**) 1100 °C. The labels of A–I and A’–I’ term places of chemical analysis stated in Table 1.

The high amount of unreacted nickel and titanium particles layered with the Ti_2_Ni phase and nickel aluminides remains in the samples with contents of aluminum up to 3 wt % at the sintering temperature of 800 °C. The aluminum content is low. Therefore, only areas with higher content of aluminum (labelled C in Figure 10) were observed. The microstructures of binary NiTi46, ternary NiTiAl1, NiTiAl3, NiTiAl5, and NiTiAl7 samples sintered at 800 and 1100 °C are shown in Figure 10. It is possible to observe that high sintering temperatures support formation of individual phases and hence, at higher sintering temperatures, there are larger areas of phases due to easier diffusional reaction.

The NiTi phase matrix without the unreacted titanium and nickel particles was formed at the sintering temperature of 800 °C, when the reaction mixture contained over 5 wt % of aluminum. The Ni_4_Ti_3_ phase was formed in the NiTiAl5 samples sintered at both temperatures according to XRD analysis. This phase is a precursor for the formation of some aluminides phase, probably for the AlNi_2_Ti phase. The microstructures of the samples with the highest addition of aluminum NiTiAl7 are similar, only the diffusion at the higher temperature of sintering enables to create the coarser grain microstructure. The area fraction of the Ti_2_Ni phase increases with the increasing aluminum content in the fully-reacted samples. The samples sintered at the lower temperature contain more Ti_2_Ni phase in comparison with the NiTiAl5 and NiTiAl7 samples, see in Table 1. The chemical composition of the individual phases determined by EDS is summarized in Table 1. The content of 5 wt % of aluminum is the trigger for formation of new phases with high amounts of aluminum in microstructures. 

**Figure 11:**
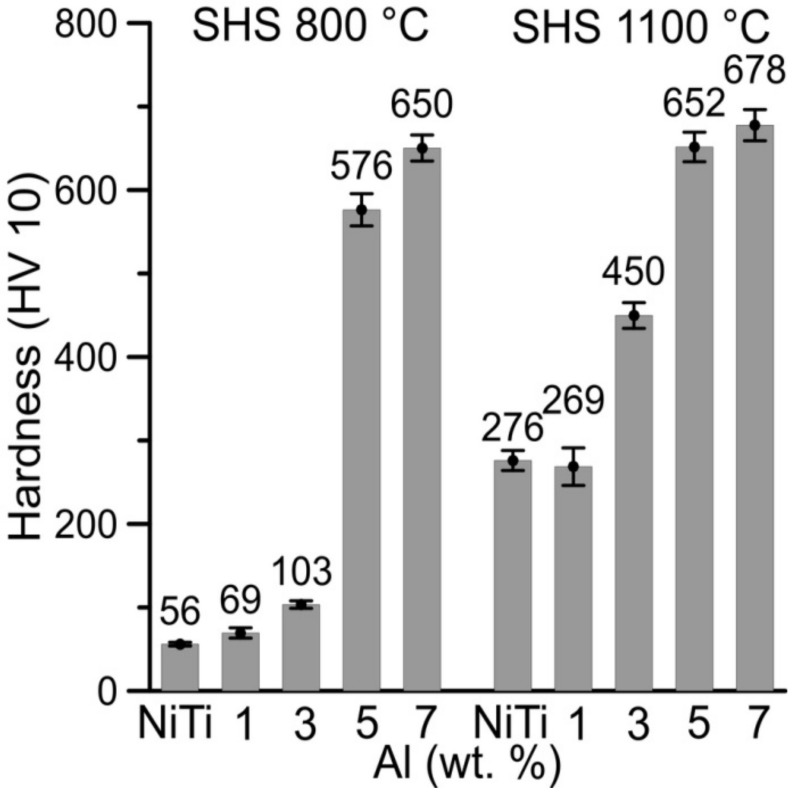
Hardness of samples sintered at 800 and 1100 °C.

The influence of aluminum on hardness and sinterability is presented in Figure 11. The lower hardness was measured in partially sintered samples of NiTi46, NiTiAl1, and NiTiAl3 at 800 °C in comparison with the sintering temperature of 1100 °C. The increasing amount of aluminum increases the hardness of the samples sintered at 800 °C; because aluminum can react larger volumes of samples, more liquid phase was formed, and the powders’ boundaries are better interconnected. This is confirmed by hardness measurement when the hardness of partially reacted powder mixture at the temperature of 800 °C increases with growing aluminum amount in the samples. The difference of hardness by using two sintering temperatures is also observed by higher aluminum content (5 and 7 wt %), but the difference in hardness decreases with the increasing aluminum addition into the samples.

The reaction mechanism was confirmed by the comparison with study [15] in which higher amount of aluminum was added into the Ni-Ti-Al powder mixture. The initial reactions are connected with the formation of nickel aluminides (Ni_2_Al_3_ and NiAl_3_) and the melt can be formed due to released heat from these reactions, even though the initiation temperature is below the eutectic temperature. The released heat is possible to measure since it significantly increases at 1 wt % of aluminum in powder mixture. The eutectic melt leads to acceleration of the occurring diffusional reaction (Ti_2_Ni phase and nickel and titanium aluminides) up to the temperature of TE-SHS between nickel and titanium at 942 °C. These reactions are studied under the conditions of lower heating rate, for example 20 °C·min^−1^ in [10]. The high heating rates (100 °C·min^−1^ in induction furnace and higher heating rate of 300 °C·min^−1^ achieved by inserting of samples into preheated electric resistance furnace) lead to TE-SHS at the temperature under the eutectic point in the Ni-Ti binary system and bellow the temperature 882–890 °C stated in [7].

## 3. Materials and Methods 

The metallic powders with the following particle sizes and purities were used as reagents: nickel (<150 µm; 99.99 wt %), titanium (<44 µm; 99.5 wt %) and aluminum (<44 µm; 99.7 wt %). The powder mixtures were blended in desired proportions (shown in Table 2) and uniaxially compressed at room temperature to cylindrical green bodies of 12 mm in diameter at a pressure of 450 MPa for five minutes using LabTest 5.250SP1-VM universal loading machine (Labortech, Opava, Czech republic). The differential thermal analysis (DTA) of the Ni-Ti-Al compressed powder mixtures was carried out using the SETSYS Evolution–1750 device (Setaram, Caluire, France) by the heating rate ranging from 50 to 1200 °C with the heating rate of 30 °C·min^−1^ in argon atmosphere and alumina crucible. The weight of the tested samples was approximately 80 mg, which was mechanically separated from the compressed powder mixture. The substitution for the DTA with a maximal applicable heating rate of 30 °C·min^−1^ was searched for measuring and observing of influence of heating rate on the initial temperature of TE-SHS reaction. The device composed of induction furnace with the protective argon atmosphere and optical pyrometer OPTRIS P20 2M (Optris, Portsmouth, NH, USA) can record the heating curve with the initial temperature at a heating rate of approximately 110 °C·min^−1^. 

The TE-SHS reaction of the compressed powder mixtures was performed in the fused silica ampoules evacuated to 10^−2^ Pa and sealed. The ampoules with the samples was inserted to the electric resistance furnace preheated to the temperature of 800 and 1100 °C for a process duration 20 min followed by cooling in air. The NiTiAl5 sample was annealed at 610 °C for 20 min to determine which phase is formed as the first. The metallographic samples were ground by sandpapers P180–P4000 (Hermes Schleifmittel GmbH, Hamburg, Germany) with SiC abrasive elements, polished by diamond pastes and etched in Kroll’s reagent (10 mL HF, 5 mL HNO_3_, and 85 mL H_2_O). The microstructure was observed by the scanning electron microscope TESCAN VEGA 3 LMU (Tescan, Brno, Czech republic) equipped with the OXFORD Instruments X-max 20 mm^2^ SDD EDS analyser (Oxford Instruments, High Wycombe, UK) for identification of the chemical composition of the individual phases. The phase composition of the alloys was identified on the samples’ ground surfaces of using X-ray diffraction (XRD) by the means of PANalytical X’Pert Pro diffractometer (PANalytical, Almeo, The Netherlands) with a copper anode. The Vickers hardness test with a load of 10 kg at laboratory temperature was performed in order to compare the quality of sintering at temperatures of 800 and 1100 °C.

## 4. Conclusions

This investigation describes the differences in the reactive sintering mechanism between the binary NiTi46 and the powder mixture with various additions of aluminum NiTiAl1–7 wt %. The changes in sintering duration were observed when the addition of aluminum into NiTi powder mixture was higher than 5 wt %. The diffusion-controlled reaction with the Ti_2_Ni phase as a product occurs below the α→β Ti transformation temperature (882 °C) and close to this temperature (860–890 °C) TE-SHS is initiated in NiTi46, NiTiAl1, and NiTiAl3 wt % powder mixtures. The aluminum content 5–7 wt % in powder mixture activates the TE-SHS at approximately 630–640 °C by heating rate 110 °C·min^−1^ and higher. The microstructures of the samples sintered at the temperatures of 800 and 1100 °C are very similar in the case of NiTiAl7 alloys.

## Figures and Tables

**Table 1:** Phase compositions and chemical compositions of individual phases of samples sintered at 800 and 1100 °C.

Sample	Area	Ni (wt %)	Ti (wt %)	Al (wt %)	Area fraction of Ti_2_Ni phase (%)
NiTi46 800°C	NiTi	51.3 ± 0.25	48.7 ± 0.25	0.0	-
	Ti_2_Ni	36.2 ± 0.32	63.8 ± 0.32	0.0	
	Ni_3_Ti	75.9 ± 0.54	24.1 ± 0.54	0.0	
NiTi46 1100 °C	NiTi’	54.2 ± 0.08	45.8 ± 0.08	0.0	11.7 ± 0.74
	Ti_2_Ni’	37.4 ± 0.08	62.6 ± 0.08	0.0	
NiTiAl1 800 °C	NiTi	52.0 ± 0.50	48.0 ± 0.50	0.0	-
	Ti_2_Ni	36.1 ± 0.43	63.9 ± 0.43	0.0	
NiTiAl1 1100 °C	NiTi	54.1 ± 0.18	45.0 ± 0.21	0.9 ± 0.05	14.7 ± 0.91
	Ti_2_Ni	39.4 ± 0.32	60.2 ± 0.26	0.4 ± 0.08	
NiTiAl3 800 °C	A	53.7 ± 1.15	46.2 ± 1.15	0.0	-
	B (Ti_2_Ni)	37.2 ± 0.35	62.8 ± 0.35	0.0	
	C	82.0 ± 3.55	7.2 ± 5.95	10.8 ± 2.54	
NiTiAl3 1100 °C	A’	53.5 ± 0.10	43.4 ± 0.10	3.1 ± 0.04	17.1 ± 1.73
	B’ (Ti_2_Ni)	37.5 ± 0.27	59.7 ± 0.26	2.8 ± 0.05	
NiTiAl5 800 °C	D	54.4 ± 0.82	41.2 ± 0.94	4.4 ± 0.19	36.2 ± 1.08
	E	38.1 ± 0.37	57.7 ± 0.50	4.2 ± 0.21	
NiTiAl5 1100 °C	D’	53.2 ± 0.14	42.5 ± 0.15	4.3 ± 0.11	24.4 ± 1.41
	E’ (Ti_2_Ni)	37.7 ± 0.50	58.8 ± 0.72	3.5 ± 0.24	
	F'	58.7 ± 0.08	30.1 ± 0.14	11.2 ± 0.13	
NiTiAl7 800 °C	G	54.7 ± 0.34	40.3 ± 0.37	5.0 ± 0.12	42.8 ± 2.07
	H (Ti_2_Ni)	38.6 ± 0.39	55.9 ± 0.46	5.5 ± 0.19	
	I	58.3 ± 0.20	30.4 ± 0.21	11.3 ± 0.15	
NiTiAl7 1100 °C	G’	53.8 ± 0.17	42.0 ± 0.14	4.2 ± 0.05	36.0 ± 2.23
	H’ (Ti_2_Ni)	37.8 ± 0.29	57.8 ± 0.83	4.4 ± 0.56	
	I'	58.9 ± 0.14	30.0 ± 0.12	11.1 ± 0.05	

**Table 2:** Chemical compositions of the prepared powder mixtures.

Sample	Chemical Composition (in wt %)		
	Ni	Ti	Al
NiTi46	54.0	46.0	-
NiTiAl1	53.5	45.5	1.0
NiTiAl3	52.4	44.6	3.0
NiTiAl5	51.3	43.7	5.0
NiTiAl7	50.2	42.8	7.0
